# Gender-Specific Covariations between Competencies, Interest and Effort during Science Learning in Virtual Environments

**DOI:** 10.3389/fpsyg.2017.01681

**Published:** 2017-10-24

**Authors:** Eva Christophel, Wolfgang Schnotz

**Affiliations:** Department of Psychology, University of Koblenz and Landau, Landau, Germany

**Keywords:** virtual science learning environments, situational interest, mental effort, arithmetic-operative competences, strategic competences, facilitating function, enabling function, gender

## Abstract

Women are still underrepresented in engineering courses although some German universities offer separate women’s engineering courses which include virtual STEM learning environments. To outline information about fundamental aspects relevant for virtual STEM learning, one has to reveal which similarities both genders in virtual learning show. Moreover, the question arises as to whether there are in fact differences in the virtual science learning of female and male learners. Working with virtual STEM learning environments requires strategic and arithmetic-operative competences. Even if we assume that female and male learners have similar competences levels, their correlational pattern of competences, motivational variables, and invested effort during virtual STEM learning might differ. If such gender differences in the correlations between cognitive and motivational variables and learning behavior were revealed, it would be possible to finetune study conditions for female students in a separate engineering course and shape virtual STEM learning in a more gender-appropriate manner. That might support an increase in the number of women in engineering courses. To reveal the differences and similarities between female and male learners, a field study was conducted with 56 students (female = 27, male = 29) as part of the *Open MINT Labs* project (the German term for Open STEM Labs, OML). The participants had to complete a virtual STEM learning environment during their regular science lessons. The data were collected with questionnaires. The results revealed that the strategic competences of both genders were positively correlated with situational interest in the virtual learning environment. This result shows the big impact strategic competences have for both genders regarding their situational interest. In contrast, the correlations between mental effort and competences differed between female and male participants. Especially female learners’ mental effort decreased if they had more strategic competences. On the other hand, female learners’ mental effort increased if they had more arithmetic-operative competences. All in all, female learners seem to be more sensitive to differences in their strategic and arithmetic-operative competences regarding their mental effort. These results imply that the implementation of separate women’s engineering courses could be an interesting approach.

## Introduction

Students differ in their preferences for specific subjects and these differences seem to be partly correlated with gender (Buse and Bilimoria, 2014). In Germany, for example, only 21 percent of female student beginners in 2014 were enrolled in STEM courses to obtain an engineering position (Statistisches Bundesamt, 2016). On the other hand, 44 percent of student beginners in chemistry and 48 percent of student beginners in mathematics were female (Kompetenzzentrum Technik-Diversity-Chancengleichheit e.V, 2015). Indeed, various studies have found that boys have somewhat higher spatial skills than girls, whereas girls have somewhat higher verbal skills and read slightly better than boys (Halpern, 2012). However, recent research revealed that such cognitive differences changing; moreover, the data depending on different tasks characteristics (Miller and Halpern, 2014). Additionally, recent research has indicated that such an essentialist view of social categories such as gender is highly problematic as it presupposes the existence of natural, clear-cut categories with relatively time-stable properties. There is little support for this essentialist view, especially from the side of neuroscience. It has proved difficult to replicate studies on gender differences in the functional organization of brain regions related to specific cognitive skills, which implies that research findings on this issue need to be reflected very carefully (Rippon et al., 2014). Contrary to the assumption of essential differences, Hyde recently concluded from a meta-analysis of gender-specific studies that “male and females are similar on most, but not all psychological variables” (Hyde, 2005, p. 581); this resulted in the formulation of a gender similarity hypothesis.

Despite these gender similarities, the question still arises as to which reasons might influence women’s and men’s different study choices. One possible explanation could be gender-specific socialization differences. From an antiquated point of view, gendered socialization means that women and men grow into society with fixed role labels, upon which they usually do not reflect (Gudjons, 2006). Indeed, recent perspectives of socialization describe both genders as socially structured with many inter-individual differences, but embedded in an asymmetric gender proportion (Bührmann et al., 2000). Although, as Bührmann et al. (2000) argued, the dual concept of gender-specific socialization is questionable, structural differences between genders are reflected by labor market segregation, indicating undercover mechanisms. Possible explanations might be allocative discrimination of women in engineering positions, when women with the same competencies and work-related characteristics as men are treated differently from their male counterparts (Brylla, 2005). For instance, some job advertisements can be posted only in male dominated networks. As Brylla (2005) mention, women start also frequently in lower positions and earn less money than their male colleagues. Further possible explanation could be found in different ways of learning, especially in corporate learning situations. Auszra (2001) suggested that women require specific learning formats in order to have the opportunity to learn need-orientated. On the one hand these formats should reflect women’s biographical learning requirements, fostering the compatibility of job and family. On the other hand, one could speculate whether women prefer different forms of learning than men. Samel (2000), for example, considers men’s behavior on the average as more competitive, whereas women are assumed to behave more implicitly, show more cooperative behavior and act more carefully than their male counterparts. These differences might be due to different socialization pattern of men and women. Moreover, of course, differences of that kind should not be interpreted as dichotomies but at the very most as slight tendencies because effect sizes are usually very small and the assumed gender differences are not as big as assumed (Hyde, 2005).

Regardless of the small differences and the impressive gender similarities, creating a customized learning situation for women can provide them with an opportunity to learn without the system of male dominance and gender-specific competitive situations (Auszra, 2001). For this reason, several universities have set up engineering courses that are only open to female students in Germany (Kuntz-Brunner, 2017). These courses incorporate virtual STEM learning environments that enable women to work in individualized situations on STEM contents without social competition and prevent the multifaceted mechanisms of gender discrimination from operating.

Despite the fact that female and male learners differ only marginally in competences (Hyde, 2005), there can be differences in the sensitivity of their competences in virtual science learning. For instance, the correlational pattern of cognitive competences, motivational variables, and invested effort when working with a virtual STEM learning environment might differ between female and male learners. If such gender differences were revealed with regard to correlations between cognitive and motivational variables and learning behavior, it would be possible to finetune study conditions for female students in a more gender-appropriate manner, creating a separate course for women and a virtual STEM learning environment adapted to female learners. Such motivational considerations cannot solve the problem of women’s underrepresentation in engineering courses as a whole, of course, because they represent only a minor part of the picture regarding gender-specific barriers. However, they could be one small step within a series of others toward solving the problem.

With reference to this background, the following study will determine whether there are any correlations between cognitive competences, situational interest, and the individually invested mental effort of female and male learners when learning with virtual STEM learning environments. First, we will describe the common characteristics of learning with virtual STEM learning environments. Then, we will show how the competences needed for virtual science learning might affect the learners’ situational interest and the mental effort they invest in their virtual STEM learning. After that, we will describe supposed similarities and differences between female and male students when learning with virtual STEM learning environments.

## Theory

### Virtual STEM Environments

Any kind of science learning requires students to understand the logic of experimentation, which involves the evaluation of theory-based hypotheses under controlled conditions (Diekmann et al., 2010; Drexler and Baker-Schuster, 2012). Learners have to understand that relevant conditions have to be manipulated systematically in order to investigate how experimental results change when conditions vary. Besides conceptual understanding of the presented phenomena, mathematic competence (particularly arithmetic-operative skills) plays an important role when students are required to analyze quantitative data from experiments with regard to specific regularities or hypotheses.

Computer-based STEM learning environments offer learners the possibility to perform virtual experiments that enable them to test experimental conditions with regard to their outcome and to interact with the learning content on their own in an exploratory fashion. The virtual STEM environment encompasses all the knowledge necessary to understand and carry out the experiment, the simulated experiment itself, and its evaluation. Learners can independently investigate, manipulate, and change specific conditions that are expected to affect the results, and engage in the arithmetic-operative operations that are necessary to evaluate the experiments. In other words, these environments allow active science learning in the classroom due to their interactivity because learners can interact with and react to the subject matter and monitor and control the investigation process (Wirth and Leutner, 2006; Niegemann et al., 2008).

Effective learning in computer-based experimental learning environments is affected by strategic competences, which enable learners to plan experiments in a mindful and hypothesis-driven manner based on their conceptual understanding of the subject matter, and by mathematical skills, which are required for the quantitative analysis of results. Furthermore, it might be affected by motivational aspects such as interest and effort, which therefore constitute further requirements. According to Schraw et al. (2001), situational interest can be defined as the “temporary interest that arises spontaneously due to environmental factors such as task instructions or an engaging text” (p. 211). Thus, situational interest is environmentally activated and can possibly be fostered by virtual learning environments in which learners can control experimental conditions and actively investigate the research subject in their own way.

In the following, the relational pattern between strategic competences, mathematic competence, situational interest, and mental effort invested by the learner in virtual experimental STEM environments will be analyzed more closely with the aim of uncovering possible similarities and differences between female and male learners.

### Strategic and Arithmetic-Operative Competences

Virtual STEM learning environments offer high potential for interactions. When learning with such learning environments, learners have to interact with the learning content to generate information (Wirth and Leutner, 2006). This means that learners have to plan and carry out actions; they have to record and analyze their observations, reflect on their results, and monitor their own learning process and adapt it, if necessary. As a consequence, learners need specific strategic competences to deal with the requirements of the virtual learning environment, for example, planning, analysis, monitoring, and reflection (Pokay and Blumenfeld, 1990; Heyn et al., 1994; Lin et al., 1999). Furthermore, a learner’s arithmetic-operative competences allow her or him to solve the mathematic numerical operations associated with experimentation more easily. Thus, these competences are also highly important for students’ mental effort when learning with virtual experimental STEM environments (Philipp, 2013). Hence, learners’ strategic competences can be seen as an essential requirement for using virtual STEM learning environments, dealing with the learning content, and managing the demands of such an environment.

### Situational Interest

Students’ strategic competences allow them to interact more intensively with learning content (e.g., Ballstaedt, 2006). Therefore, these competences can also foster learners’ spontaneous interest in the learning subject, as learners can explore the content more deeply when learning in a virtual learning environment. The implementation of learning strategies brings the learner closer to understanding the learning subject and its details. Indeed, the requirements of the learning situation during virtual experimentation encourage students to consistently use learning strategies, and higher usage of learning strategies (or higher strategic competence) can result in higher situational interest. As explained above, situational interest is defined as a spontaneous and environmentally activated type of interest that is based on the conditions of the learning situation and learning material (Schraw et al., 2001; Grüninger, 2013). Accordingly, virtual learning environments can be assumed to activate learners’ spontaneous interest in the learning subject, provided that the learners have the necessary strategic competences to explore the environment. Thus, we assume that students with higher strategic competences are more likely to develop situational interest because their higher competences open up a broader range of possible experimental manipulations and investigations for them compared to students with lower strategic competences. The more competences they have, the higher situational interest can be expected to be and the more deeply the environment will be explored. Consequently, the usage of strategic competences has an inner relation with the content of the virtual learning environment. In contrast, arithmetic-operative competences are not related to content. Operations with numbers in general do not have a semantic relation with learning content (Krasa and Shunkwiler, 2009). Thus, there might be no inherent relation between the learners’ arithmetic-operative competences and their situational interest. Next, we will consider the possible effects of arithmetic-operative and strategic competences on mental effort.

### Mental Effort as a Function of Competences

The relationship between competences and effort seems to be inherently ambiguous. On the one hand, learning requires the usage of limited resources such as time and working memory. In addition, the learner has to activate motivational and energetic resources. The learners’ cognitive resources invested in the learning process are the so-called ‘mental effort’ (Sweller, 2005; Sweller et al., 2011). The mental effort invested by students during learning is affected by their competences regarding the learning domain. This is because the amount of working memory capacity required for conscious control decreases with increasing expertise, due to a higher amount of automatic processing, which requires a lower amount of effort. With reference to cognitive load theory (Sweller et al., 2011), one could assume that learners with higher competences in the learning field need less mental effort than learners with lower competences. Therefore, competences have a *facilitating function* as they reduce the effective effort (Sweller et al., 1998; Sweller, 1999; Schnotz and Rasch, 2005). Learners with higher competences have higher abilities to deal with the learning demands; hence, they have to invest less mental effort in the respective learning field than learners with lower competences. Such a facilitating effect can be assumed, for example, for the learner’s mathematical competences when learning with experimental environments because the required arithmetic operations can be performed more easily on the basis of higher competences.

Moreover, an increase of competences can also enable learners to do things they were not able to do previously with lower competences. In this case, the higher competences allow them to engage in deeper and more elaborate cognitive processing, and an increase of competences is then associated with an increase of invested cognitive effort. The learners’ mental effort is related to their willingness to engage in the cognitive requirements of the learning process. Willingness to engage in the learning-relevant processes might generally be higher in students with higher competences than in students with lower competences (Paradies et al., 2009). Thus, adequate competences are a prerequisite for performing certain processes and can therefore have an *enabling function* (Sweller et al., 1998; Sweller, 1999; Schnotz and Rasch, 2005). Learners with higher competences deal better with the learning demands; hence, for them it might be easier to solve the learning tasks. As a consequence they might be willing to invest more mental effort in the respective learning field than learners with lower competences. Such an enabling effect can be assumed especially for learners’ strategic competences because learning with virtual environments encompasses a broad range of possible interactions with the learning content, including deep consideration of complexity. Higher strategic competences can increase mental effort required to complete virtual STEM learning environments.

**FIGURE 1:**
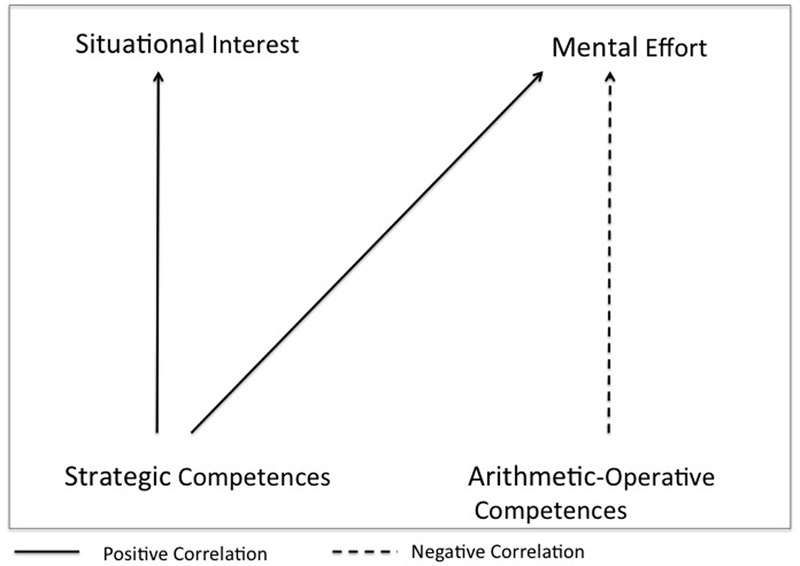
Hypothetical correlations between arithmetic-operative competences, strategic competences, situational interest, and mental effort with a focus on the facilitating function and enabling function (Sweller et al., 1998; Sweller, 1999; Schnotz and Rasch, 2005).

The general correlational pattern between conceptual competence, mathematical skills, situational interest, and mental effort expected based on these assumptions is depicted in **Figure 1**. The figure shows that we expect strategic competences to be correlated with higher situational interest, and we expect that higher arithmetic-operative competences could have a facilitating function and be correlated with lower mental effort, while strategic competences could have an enabling function and be correlated with higher mental effort (**Figure 1**).

### Similarities and Differences between Female and Male Students

Learning with virtual STEM learning environments leverages the general habits of the so-called ‘digital native’ generation regarding usage of new media. Female and male learners might be rather similar in this respect because both are familiar with these media and usually enjoy working with them (Prensky, 2001). Accordingly the assumed positive correlation between strategic competences and situational interest might be the same for both genders. However, there might also be gender differences with regard to the above-mentioned competences. Several studies have revealed that, on the one hand, female learners use more strategic competences in learning situations on average than male learners (Dresel et al., 2004; Ziegler and Dresel, 2006). On the other hand, male learners frequently have more arithmetic-operative competences than female learners (Artelt et al., 2003; Halpern, 2012). Because, as Rippon et al. (2014) emphasized, it has proved difficult to replicate studies on gender differences in the functional organization of brain regions related to specific cognitive skills, research findings on this issue need to be reflected on very carefully. If we assume, however, that higher strategic competences lead to higher willingness to use such competences (Paradies et al., 2009), we can speculate that there might be differences in female and male learners’ correlational patterns of situational interest, mental effort, and strategic and arithmetic-operative competences. Indeed, the sensitivity of women and men’s strategic and arithmetic-operative competences might differ regarding their mental effort.

## Questions And Hypotheses

The purpose of our study was to determine whether there are differences and similarities in the sensitivity of female and male learners’ arithmetic-operative and strategic competences in virtual science learning with regard to their situational interest and mental effort. If gender differences and similarities in the correlation patterns are revealed, it would be possible to finetune study conditions in a more gender-appropriate manner for female students, creating a separate course for female learners. That might support an increase in the number of women in engineering courses. With a focus on this purpose, we investigated the following research hypotheses:

### Situational Interest

(H1a) Higher strategic competences are associated with higher situational interest in a virtual learning environment.
(H1b) The correlational patterns between situational interest and strategic competences are similarly pronounced in female and male learners.

### Mental Effort

(H2a) Higher strategic competences have an enabling function regarding the conceptual planning of virtual experiments, resulting in more elaborated cognitive processing, which leads to higher invested mental effort.
(H2b) Higher arithmetic-operative competences have a facilitating function for the processing of quantitative data generated by virtual experiments. For specific computational tasks, higher arithmetic-operative competences are therefore associated with lower mental effort.
(H2c) The correlational patterns between mental effort and arithmetic-operative and strategic competences are differently pronounced in female and male learners.

## Methods

The following study is based on a project about the usage of virtual STEM labs conducted by the Universities of Applied Sciences Kaiserslautern, Koblenz, Trier, and Koblenz-Landau funded by the German Federal Ministry of Education and Research. The project aims at enabling college students of engineering to plan, conduct, and evaluate interactive virtual science experiments with a special emphasis on supporting female students in the domain of STEM (Fleuren et al., 2014).

### Participants

A total of 56 high school students from 10th and 11th grades with a mean age of 15.89 (*SD* = 2.31) participated in this study. The precondition for their participation was that their legal guardians signed a consent form, which contained information about compliance with data protection guidelines as well as about the study’s aims, procedure, and material. In the sample, 27 of the participants were female and 29 were male. The participants had a mean grade of 2.46 (*SD* = 1.05) in the relevant subject (chemistry or physics) in the previous school year.

### Variables and Materials

We used gender (female vs. male), strategic competences, and arithmetic-operative competences as independent variables and mental effort and situational interest as dependent variables. The values for *Cronbach’s Alpha* were assessed with 214 participants from all studies within the OML project (see values below).

To assess the strategic competences, we used three scales (planning, regulation, and monitoring) of the Kieler learning strategy inventory (KSI; Heyn et al., 1994) which has a four-step answer format (example item: “If I prepare myself/learn I make myself a list with the important things and learn it afterwards”). The value for *Cronbach’s Alpha* was 0.88. The arithmetic-operative competences were assessed with two task packages from the Berlin intelligence structure test (BIS 4; Jäger et al., 1997) which challenge students to solve mathematical problems in a fixed time frame, moderated by an experimental assistant (example item: “A worker earns €15.20 per hour. How much does he earn if he works 5 h?”). The value for *Cronbach’s Alpha* was 0.99. Mental effort was collected with an item of Paas et al. (1994) and an item of Wagner (2013). These items capture students’ judgment of the amount of effort required and of the task difficulty (example item: “How much did you exert yourself while solving the learning environment?”). Both items included a nine-step answer format. The value for *Cronbach’s Alpha* for mental effort was 0.56 and, therefore, low; this might be because the scale measures both estimated effort and perceived difficulty. The situational interest of students in the completion of the learning environment was explored with adapted items from the questionnaire in order to capture the actual motivation in learning and performance situations (FAM; Rheinberg et al., 2001). The value for *Cronbach’s Alpha* was 0.81. The FAM questionnaire included a seven-step answer format (example item: “In this learning environment I like the role of the scientist who discovers relationships”).

### Procedure

**FIGURE 2:**
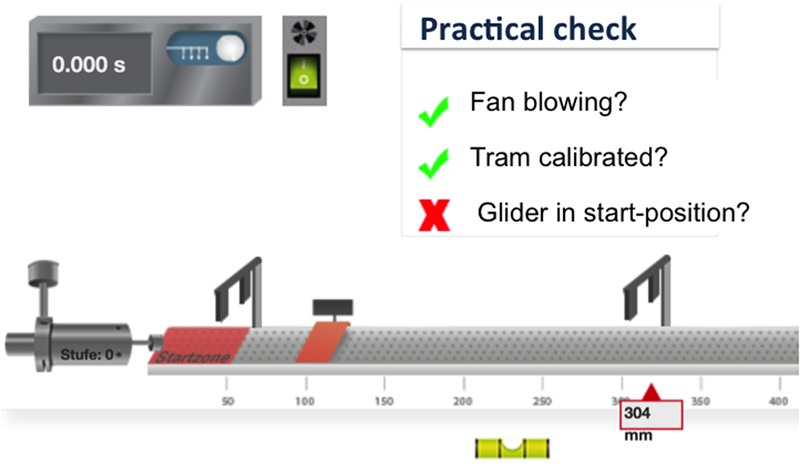
*Experiment* chapter within the virtual learning environment: *air cushion tram* (Roth et al., 2014, p. 5).

The field study was carried out in the classroom during regular school lessons of physics or chemistry. First the students had to answer two task packages from the Berlin intelligence structure test (BIS; Jäger et al., 1997) in a fixed time frame, which was moderated by an experimental assistant. In the next step the students had to complete a virtual learning environment, which had been created in the OML project and includes five chapters: orientation, foundations, experiment, application, and reflection. The *orientation* chapter offers a motivational introduction to the learning environment. Additionally, it gives an overview of the content, goals, and relevant previous knowledge. The *foundations* chapter offers an overview of the theoretical background (e.g., the content, relevant diagrams, and formulas) and relevant questions or hypotheses. In the *experiment* chapter, the students investigate the questions or hypotheses by trying out different virtual experimental tools. **Figure 2** shows an example from the virtual learning environment, *air cushion tram*, which was used in physics lessons and developed at the University of Applied Sciences in Trier (Roth et al., 2014).

The *application* chapter invites students to apply the knowledge and strategies they have learned in the previous chapters. The *reflection* chapter sums up the learned content and, with several questions, encourages learners to evaluate their own learning process. The virtual learning environment is interactive, enabling students to switch between different chapters. Thus, the learners can, for instance, reread the theoretical background if they have problems completing the virtual experiment or they can go back to the experiment during application of the learned content.

After completion of the learning environment, the participants received a questionnaire that assessed their mental effort, situational interest, and strategic competences (see variables and material above).

## Results

In a first step, we computed the correlations between the four variables *mental effort, situational interest, strategic competences, and arithmetic-operative competence*. **Table 1** shows that the learners’ situational interest was significantly positively correlated with their strategic competences. The participants’ arithmetic-operative competences were significantly negatively correlated with their strategic competences.

**Table 1:** Pearson correlations, means, and standard deviations from the scales: Mental Effort, Situational Interest, Strategic Competences, and Arithmetic-Operative Competences (*N* = 56).

Indicant	1	2	3	4
Mental effort				
Situational interest	0.042			
Strategic competences	-0.073	**0.239^∗^**		
Arithmetic-operative competences	0.012	0.199	-**0.226^∗^**	
Mean	10.45	11.71	47.36	5.78
*SD*	3.75	5.37	12.34	1.90

*^∗^*p* < 0.05, ^∗∗^*p* < 0.01, one-tailed.*

In a next step, we examined the female and male learners’ differences for the variables *mental effort, situational interest, strategic competences, and arithmetic-operative competences*. The results are displayed in **Table 2**.

**Table 2:** Means, standard deviations and difference in means of both genders of the scales *Mental Effort, Situational Interest, Strategic Competences, and Arithmetic-Operative Competences* of Female and Male Participants.

	Female		Male		Total
*N*	27		29		56
Variable	Mean	*SD*	Mean	*SD*	Difference in means of both genders
Mental effort	10.70	4.11	10.21	3.44	0.49
Situational interest	10.89	4.75	12.48	5.86	1.59
Strategic competences	51.37	11.63	43.62	11.98	**7.75^∗^**
Arithmetic-operative competences	5.22	1.53	6.28	2.09	**1.06^∗^**

*Results of *t*-tests, ^∗^*p* < 0.05, ^∗∗^*p* < 0.01.*

To test our hypotheses, we conducted regression analyses with strategic competences, gender (female vs. male), and the interaction of these two factors as predictors and with mental effort and situational interest as the respective criteria.

### Situational Interest

To verify Hypotheses1a and 1b, we ran regression analyses with the variables situational interest, strategic competence, and gender. The model with the predictors gender (female vs. male) and strategic competences significantly predicted situational interest, *R*^2^ = 0.11, *adj R*^2^ = 0.08, *F*(2,53) = 3.34, *p* = 0.041. The model revealed that situational interest was affected by students’ strategic competences, β = 0.32, *t*(55) = 2.34, *p* = 0.023. This indicates that students with more strategic competences had more situational interest in the virtual learning environment (**Table 3**). Furthermore, gender had no impact on situational interest, β = -0.25, *t*(55) = -1.84, *p* = 0.072. Accordingly, there was no difference between the situational interest of female and male participants in the virtual learning environment. In the next step, we included interaction between gender and strategic competences in the model, *R*^2^ = 0.16, *adj R*^2^ = 0.12, *F*(3,52) = 3.38, *p* = 0.025. However, the interaction effect was not significant, β = -0.22, *t*(55) = -1.75, *p* = 0.086.

**Table 3:** Hierarchical regression predicting situational interest (*N* = 56).

Variable	*B*	*SE(B)*	β
**Step 1**			
Gender	-1.38	0.75	-0.25
Strategic competences	0.14	0.06	**0.32^∗^**
**Step 2**			
Gender x strategic competences	-0.11	0.06	-0.22

*Strategic competences were centered. Effect coding was used for gender. ^∗^*p* < 0.05, ^∗∗^*p* < 0.01, *R*^*2*^ = 0.16, *adj R*^*2*^ = 0.12, *F*(3,52) = 3.38, *p* = 0.025.*

The model with the predictors gender and arithmetic-operative competences and the criterion situational interest was not significant, *R*^2^ = 0.05, *adj R*^2^ = 0.01, *F*(2,53) = 1.37, *p* = 0.263. In the next step, we included interaction between gender and arithmetic-operative competences in the model, but neither the model, *R*^2^ = 0.05, *adj R*^2^ = -0.01, *F*(3,52) = 0.90, *p* = 0.447, nor the interaction effect, β = 0.02, *t*(55) = 0.13, *p* = 0.897, was significant.

To sum up, in accordance with our Hypothesis 1a, the results show that strategic competences are positively correlated with students’ situational interest. In accordance with our Hypothesis 1b, this correlation did not differ between male and female participants. Additionally, there was no correlation between situational interest and arithmetic-operative competences.

### Mental Effort

To test Hypotheses 2a and 2c, we ran regression analyses with the variables *mental effort, strategic competences*, and *gender*. The model with the predictors gender (female vs. male) and strategic competences and the criterion mental effort was not significant, *R*^2^ = 0.01, *adj R*^2^ = -0.02, *F*(2,53) = 0.39, *p* = 0.681. In the next step, we included interaction between gender and strategic competences in the model, which was then significant, *R*^2^ = 0.15, adj *R*^2^ = 0.11, *F*(3,52) = 3.16, *p* = 0.032. In addition, interaction was significant, β = -0.37, *t*(55) = -2.93, *p* = 0.005, while the regression coefficient for gender (β = 0.11, *t*(55) = 0.84, *p* = 0.405) and the regression coefficient for strategic competences (β = -0.12, *t*(55) = -0.87, *p* = 0.387) were not significant; strategic competences showed no enabling function for mental effort for both genders.

**FIGURE 3:**
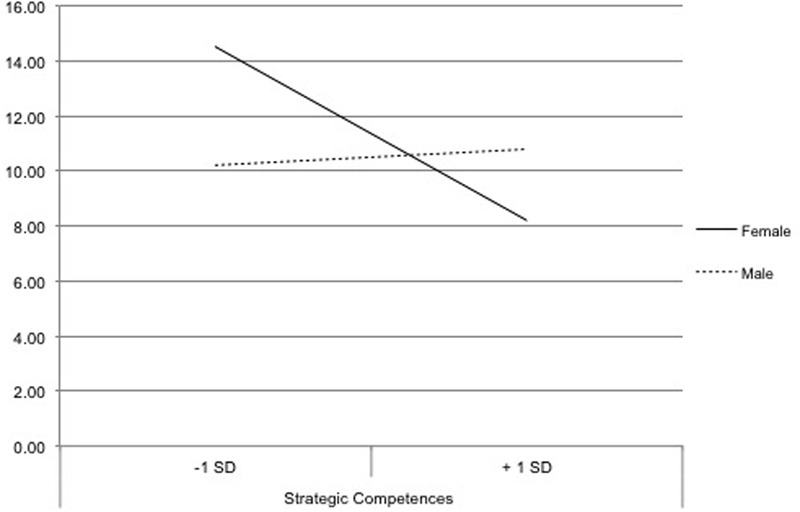
Interaction effect between gender and strategic competences on participants’ mental effort.

To verify Hypothesis 2c, we interpreted the interaction effect. Therefore, we used dummy coding for the dichotomous variable (0 = female/1 = male; 1 = female/0 = male) and included it in two complementary regression models. In the first model, in which the female group was coded 0 and the male group was coded 1, the regression coefficient for strategic competences was significant, *B* = 0.24, *t*(55) = 2.93, *p* = 0.005. Thus, strategic competences indeed affected mental effort in the group of female participants. By contrast, in the second model with the opposite coding pattern, the regression coefficient for strategic competences was not significant, *B* = 0.08, *t*(55) = 1.43, *p* = 0.158. Thus, higher strategic competences decreased the mental effort of female participants, whereas higher strategic competences had no effect on the mental effort of male participants. Consequently, strategic competences can be assumed to have a facilitating function, especially for females. To interpret the differences between individuals with higher (+ 1 standard deviation) and lower strategic competences (-1 standard deviation), two regression models were computed concerning mental effort (Richter, 2006). In the regression model that contained the variable indicating higher strategic competences, the coefficient for gender was not significant, *B* = 2.13, *t*(55) = 1.52, *p* = 0.135. By contrast, in the model with the variable that indicated lower strategic competences, the coefficient for gender was significant, *B* = -3.81, *t*(55) = -3.81, *p* = 0.011. Accordingly, the mental effort of female and male participants was significantly different if they had lower strategic competences. By contrast, female and male participants with higher strategic competences showed no significant difference in mental effort (**Figure 3**).

To test Hypotheses 2b and 2c, we ran a regression model with the predictors gender (female vs. male) and arithmetic-operative competences and the criterion mental effort; it was not significant, *R*^2^ = 0.01, *adj R*^2^ = -0.03, *F*(2,53) = 0.15, *p* = 0.864. In a next step, we included interaction between gender and arithmetic-operative competences in the model, which was then significant, *R*^2^ = 0.14, *adj R*^2^ = 0.09, *F*(3,52) = 2.90, *p* = 0.043. In addition, interaction was significant, β = 0.39, *t*(55) = 2.89, *p* = 0.006. Neither the regression coefficient of gender (β = 0.12, *t*(55) = 0.88, *p* = 0.381) nor the regression coefficient of arithmetic-operative competences (β = 0.16, *t*(55) = 1.12, *p* = 0.270) was significant. Consequently, gender and arithmetic-operative competences had no impact on the mental effort of participants. Hence, arithmetic-operative competences show no facilitating function with regard to mental effort generally.

**FIGURE 4:**
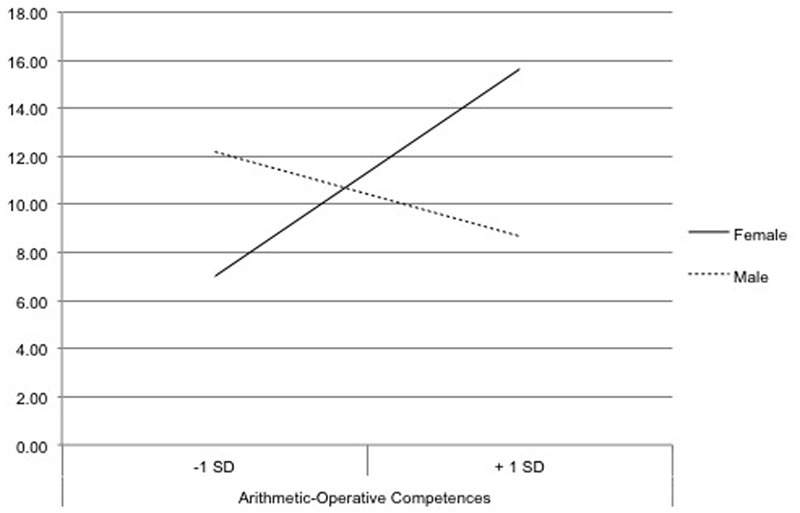
Interaction effect between gender and arithmetic-operative competences on the participants’ mental effort.

To verify Hypothesis 2c, we interpreted the interaction effect. Therefore, we used dummy coding for the dichotomous variable (0 = female/1 = male; 1 = female/0 = male) and included it in two complementary regression models. In the first model, in which the female group was coded 0 and the male group was coded 1, the regression coefficient for arithmetic-operative competences was significant, *B* = 0.58, *t*(55) = 2.51, *p* = 0.015. By contrast, in the model in which the male group was coded with 0 and the female group was coded with 1, the regression coefficient for arithmetic-operative competences was not significant, *B* = -0.24, *t*(55) = -1.46, *p* = 0.149. Thus, especially the mental effort of female participants increased if they had higher arithmetic-operative competences. Consequently, especially in the group of female participants, the arithmetic-operative competences had an enabling function. To interpret the differences between individuals with high (+1 standard deviation) and low (-1 standard deviation) arithmetic-operative competences, two regression models were computed with regard to mental effort. In the regression model that contained the variable indicating higher arithmetic-operative competences, the coefficient for gender was significant, *B* = -3.96, *t*(55) = -2.58, *p* = 0.013. By contrast, in the model with the variable that indicated lower arithmetic-operative competences, the coefficient for gender was not significant, *B* = 2.20, *t*(55) = 1.59, *p* = 0.117. Thus, the mental effort of female and male participants was significantly different if they had higher arithmetic-operative competences. If female participants had higher arithmetic-operative competences than male participants, they invested significantly more effort in learning with the virtual environment than their male counterparts. By contrast, female and male participants with lower arithmetic-operative competences showed no significant difference in mental effort (**Figure 4**).

The results of both regression models with the effect of different competences (i.e., strategic and arithmetic-operative) on mental effort are shown in **Table 4**.

**Table 4:** Regression models predicting mental effort (*N* = 56).

	Models					
	Strategic competences			Arithmetic-operative competences		
Variable	*B*	*SE(B)*	β	*B*	*SE(B)*	β
Gender	0.44	0.52	0.11	0.49	0.52	0.12
Competences	-0.04	0.04	-0.12	0.31	0.28	0.16
Gender x competences	-0.012	0.04	-**0.37^∗^**	0.84	0.29	**0.39^∗∗^**
*R*^2^		0.15			0.14	
*F*		**3.16^∗^**			**2.90^∗^**	

*Competences were centered; effect coding was used for gender, ^∗^*p* < 0.05, ^∗∗^*p* < 0.01.*

To summarize, strategic competences did not have an enabling function on metal effort in the entire sample, thus not supporting Hypothesis 2a. Additionally, arithmetic-operative competences did not have a facilitating function on the mental effort of all participants, contrary to Hypothesis 2b. In line with Hypothesis 2c, however, the correlation patterns of female and male learners show differences. The mental effort of female participants decreased if they had higher strategic competences. Thus, female learners’ strategic competences seem to have a facilitating function on mental effort. Especially female participants increased their mental effort if they had higher arithmetic-operative competences. However, contrary to Hypothesis 2b, female learners’ arithmetic-operative competences seem to have an enabling function on mental effort.

## Discussion

The mental effort and the situational interest of men and women were similar in learning with virtual science environments. The results of the present study indicate furthermore that both female and male students with higher strategic competences are more likely to show higher situational interest than students with lower strategic competences when learning from virtual STEM environments. One can therefore assume that both female and male students with higher strategic competences will engage in deeper exploration of the subject matter due to their higher spontaneous interest than students with lower strategic competences. For this reason, the use of virtual STEM learning environments can be beneficial for both genders regarding their situational interest. Because situational interest “often precedes and facilitates the development of personal interest” (Schraw et al., 2001, p. 211), both female and male students with higher strategic competences might also be more likely to develop personal interest in STEM topics than students with lower strategic competences. It follows that the use of virtual STEM learning environments in engineering courses should foster both women’s and men’s enrollment in such courses under the condition that their strategic competences are sufficiently high. Thus, in order to foster situational interest in virtual STEM learning the strategic competences of female and male students in learning with such learning environments should be enhanced.

Besides these similarities, there seem to be also differences between genders regarding the use of such learning environments. The results of the present study suggest that female students learning with virtual STEM learning environments are more sensitive to differences in strategic and arithmetic-operative competences than their male counterparts. Nevertheless, the empirical correlational patterns differed from our hypotheses. On the one hand, strategic competences seem to have a facilitating function for female students. Thus, higher strategic competences were correlated with lower mental effort (Sweller et al., 1998; Sweller, 1999). On the other hand, higher arithmetic-operative competences seem to have an enabling function for female students (Schnotz and Rasch, 2005). Accordingly, higher arithmetic-operative competences were correlated with higher mental effort. Thus, female students with higher arithmetic-operative competences were more likely to invest higher mental effort in learning with virtual STEM environments than female students with lower arithmetic-operative competences. Therefore, if female participants had more arithmetic-operative competences than male participants, they invested significantly more effort into learning with the virtual environment than their male counterparts, who’s invested effort, in contrast, was not significantly affected by arithmetic-operative competences. It seems that male learners with higher arithmetic-operative competences are more likely at risk to invest too less effort into learning than females. Further research is needed on this issue.

The use of cognitive prompts that activate and support the application of strategic competences could therefore be especially useful for female students learning with virtual experimental STEM learning environments (Lin et al., 1999). In addition to activating their strategic competences, such prompts could also increase their situational interest in the environment and learning topic. In short, to promote female students’ virtual STEM learning, they should be encouraged to use their strategic competences while working with such learning environments. More importantly, regarding female and male learners’ situational interest such prompting while virtual science learning might be a benefit for both genders. Following the cognitive apprenticeship approach, such prompts could be given systematically by the learning environment at the beginning of learning with subsequent fading out as the learner becomes increasingly proficient in operating the environment (Collins et al., 1991). Another possibility would be to insert virtual coaching into the learning environment which could include hints for carrying out the virtual experiments.

The learning of female students from virtual experimental STEM environments could also be enhanced through support of their arithmetic-operative competences and especially through their meta-cognitive awareness of these competences (Hacker et al., 1998). The enabling function of these competences means that they are likely to increase female students’ readiness to invest more mental effort in learning from experimental STEM environments.

Based on the differences found in the correlational patterns in this study, one could also tentatively consider whether it might be fruitful to create special engineering courses for women. Such courses would focus more on women’s inter-individual competence differences and promote them depending on their learning needs. Within such courses, virtual STEM learning environments could offer individualized learning situations which take into account the higher sensitivity of female students’ competences with regard to their invested mental effort and provide capabilities for appropriate support for them. With regard to the results of our study such support should especially trigger the female learners’ strategic competences. That might have a facilitating function on the female learners’ mental effort; in this case, single sex education can support the decrease of effective effort and facilitate the female learners’ sciences learning process (Sweller et al., 1998, 2011; Sweller, 1999; Schnotz and Rasch, 2005). Furthermore, the integration of such learning environments can foster the compatibility of job and family, because virtual STEM environments can be solved in individual time slots.

Albeit, one has to keep in mind that there are not dichotomous groups of men and women, but rather several individuals with, sometimes different, sometimes similar, learning-needs. In addition, one needs to understand how biological factors interact with environmental factors to maximize the benefit of virtual STEM environments (Miller and Halpern, 2014). It should be noted, furthermore, that the present findings should be interpreted with care. First, only a relatively small number of students participated in this study. Second, background information about gender socialization and gender identity was not taken into account. Further research is needed to obtain a clearer picture of the sometimes subtle differences of female compared to male learners working in STEM environments.

Motivational considerations might be one facet of the complex picture of women’s underrepresentation in engineering courses but, of course, they are far from solving the problem by themselves. For instance, they cannot solve the problem of allocative discrimination of women in engineering positions (Brylla, 2005), and they cannot remove, gender-specific socialization patterns in our society (Bührmann et al., 2000). Instead, they should be considered as a small step within a series of other research steps toward solving the problem of enhancing women’s representation in the engineering labor market.

## Ethics Statement

A full ethical review was not required for this type of study in Germany; however, the Aufsichts- und Dienstleistungsdirektion of Rheinland-Pfalz gave their permission for the study after consideration of the ethical aspects. Written informed consent was obtained from the legal guardians of all children included in the study.

## Author Contributions

EC: Substantial contributions to the conception and design of the work and the acquisition, analysis, and interpretation of data for the work; Drafting the work; Final approval of the version to be published. WS: Substantial contributions to the conception and design of work and revising it critically for important intellectual content; Final approval of the version to be published. EC and WS: Agreement to be accountable for all aspects of the work in ensuring that questions related to the accuracy or integrity of any part of the work are appropriately investigated and resolved.

## Conflict of Interest Statement

The authors declare that the research was conducted in the absence of any commercial or financial relationships that could be construed as a potential conflict of interest.
